# Rhinoscleroma: Case Report

**DOI:** 10.1016/S1808-8694(15)31006-5

**Published:** 2015-10-19

**Authors:** Mônica Elisabeth Simons, Lidio Granato, Roberto Claudio Batista Oliveira, Mônica Porto Alves Alcantara

**Affiliations:** a MD, Otorhinolaryngology Intern - Santa Casa de São Paulo; b Assistant Professor - Department of Otrhinolaryngology - Santa Casa de Misericórdia de São Paulo; c MD, Otorhinolaryngologist; d MD, 3rd Year Resident - Santa Casa de Misericórdia de São Paulo

**Keywords:** klebsiella rhinoscleromatis, microscopy, rhinoscleroma

## Abstract

Rhinoscleroma is a chronic, granulomatous infection that most frequently affects the respiratory mucosa, especially the nasal cavity and eventually extending through the lower respiratory tract. The disease is endemic in some countries of Central America (El Salvador and Guatemala), Indonesia, India, Poland, Hungary, Russia and some African countries as well. It is a rare disease in South America. We report a 51-year-old male resident of a psychiatric institution in São Paulo presenting with progressive nasal obstruction, frontal headache, yellowish nasal discharge and a mass extruding through the right nasal vestibule. The present case report describes a Rhino-Sinus scleroma where histopathology was vital in the diagnosis. The patient was treated by surgical excision of the nasal mass followed by a course of ciprofloxacin. He has remained asymptomatic up to the last visit six months following treatment and has shown no evidence of recurrence.

## INTRODUCTION

Rhinoscleroma is a chronic granulomatous, slowly progressive infection that affects the nose and other respiratory tract structures^1^. It was first described in 1870 by the dermatologist Ferdinando Von Hebra^2^ and posteriorly named respiratory scleroma. The Greek name “skleroma,” meaning hard tumefaction, was adopted in 1932 at the International Clinical Otorhinolaryngology Conference (in Madrid), emphasizing involvement of upper and lower airways^1^, ^3^. It occurs frequently in the nasal fossae, eventually extending itself to the larynx, the rhinopharynx, the mouth and the paranasal sinuses; the lips, trachea and bronchi may also be affected to a lesser degree^4^, ^5^. Extra-respiratory involvement has rarely been described^1^.

Scleroma is endemic in some Central American countries (El Salvador and Republic of Guatemala), Indonesia, India, Poland, Hungary, Russia and some African countries^1^, ^6^. In South America the frequency is low^1^, ^7^.

The first observation of scleroma published in Brazil was made by Adolpho Lutz in 1890^8^. Few cases have been reported in Brazilian medical literature, thus the reason for publishing this case, which includes the histopathology and treatment.

## CLINICAL CASE PRESENTATION

J.P.S is a 51-year-old brown unmarried male patient born in Minas Gerais, coming from Sao Paulo (SP state), and living in a psychiatric support institution. He was brought to the Otorhinolaryngology unit at the Sao Paulo Santa Casa de Misericordia Hospital by his sister, and presented a reddish mass in his right nare, with frequent bleeding episodes, for the past five months. He also had a long history of sneezing, nasal pruritus, thickened and yellowish rhinorrhea, bilateral nasal obstruction, and daily intense frontal headaches.

On further investigation, he had lost some weight (three kilograms) during those five months, and was an ex-smoker and ex-drinker of alcoholic beverages, having given up these habits four years ago. He had worked as a polisher, in contact with chemical products during 10 years, and had been retired due to a psychiatric condition (mental deficiency). He was taking biperiden, chlorpromazine and haloperidol, and lived in a psychiatric support institution.

The otorhinolaryngological exam revealed an enlarged nose due to a violet-colored mass with small ulcers, a hardened consistency, a smooth surface, and irregular borders, which extruded from the right nare. The left nasal fossa was obstructed by a complete deviation of the nasal septum. It was not possible to introduce a nasopharyngoscope. On examination with the Glatzel mirror, both nasal fossae were not pervious.

The remaining physical exam was within normal limits. Psychiatrically the patient was confused and agitated due to mental deficiency.

**Figure 1:**
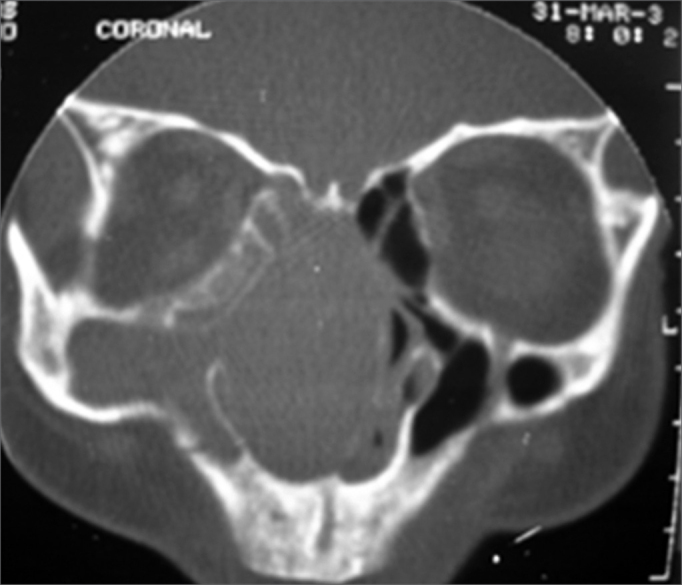
Computed tomography of the paranasal sinuses - coronal plane. Soft tissue attenuation material occupies the nasal fossae, the right maxillary sinus, and some ethmoid cells. The cartilaginous septum is deviated to the left. There is no bone destruction.

**Figure 2:**
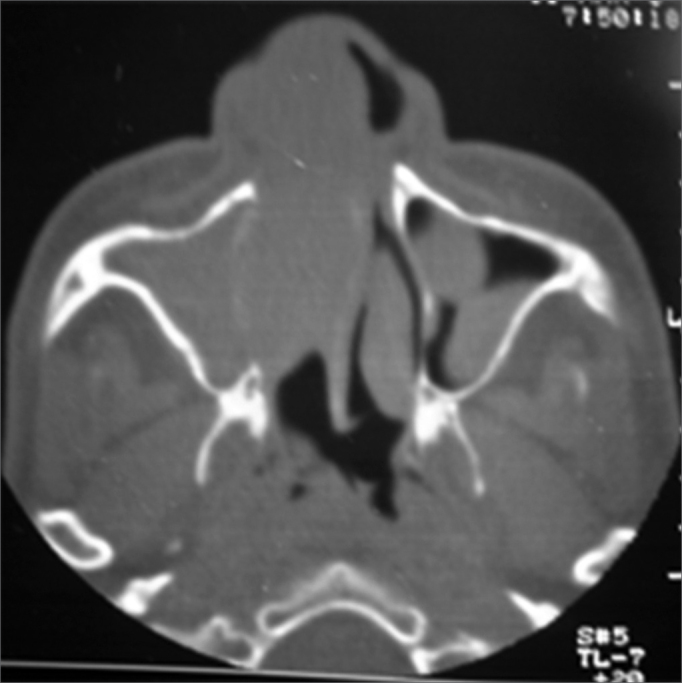
Computed tomography of the paranasal sinuses - axial plane. Soft tissue attenuation material occupies the right nasal fossa, the right maxillary sinus, and part of the left maxillary sinus. The cartilaginous septum is deviated to the left. There is no bone destruction.

Computed tomography of the paranasal sinuses revealed a lesion with soft tissue attenuation material in the nasal fossae, the right maxillary sinus, the anterior ethmoid cells, in some posterior ethmoid cells, and in part of the frontal sinus to the right. There was minor soft tissue attenuation material in the left maxillary sinus. The sphenoid sinus and the rhinopharynx were unaltered and no bone destruction was observed. (Figures 1 and 2)

Treatment included surgery followed by antibiotics. Surgical removal of the tumor from the nasal fossae was a sinusectomy through the transmaxillary approach with a degloving access route. The specimen was referred to pathology for examination. At surgery a 3 cm diameter hard whitish-gray mass was found inserted in the right maxillary sinus and the right nasal fossa, extending itself into the ethmoid sinus; it had a smooth surface with irregular contours and contained pus. Bone structures were preserved and the cartilage septum was deviated to the left by tumoral compression.

**Figure 3:**
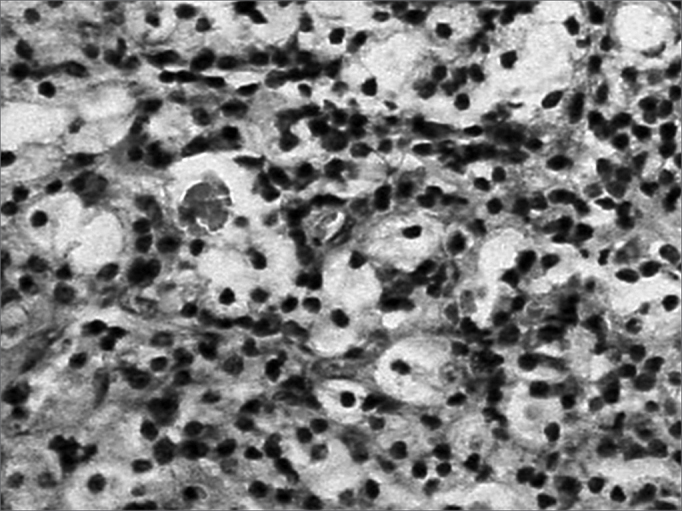
Microscopy exam of the nasal mucosa showing a lymphoplasmacytic infiltrate with Mikulicz cells, Russel bodies and congested and dilated blood vessels.

Pathology disclosed nasal mucosa containing a diffuse lymphoplasmacytic inflammatory process with large vacuolated macrophages typical of Mikulicz cells and many plasmocytes transformed into Russel bodies. (Figure 3)

Ciprofloxacin was used (500 mg. 12/12h for 14 days) based on literature data.

The patient returned to the Otorhinolaryngology unit in good physical condition and no signs of recurrence in the nasal mucosa.

## DISCUSSION

Scleroma is a specific granulomatous infection that affects the nose and, less frequently, other structures of the respiratory tract. It usually affects people with lower social and economic status, and is associated with lack of hygiene and prolonged contact with infected patients^3^, ^9^.

The disease progresses in three stages: (1) the catarrhal stage: patients have non-specific rhinitis symptoms that progress to fetid rhinorrhea, crusting and nasal obstruction. This stage can last for weeks or months. (2) the hypertrophic stage: this stage includes granulation tissue with deformity by widening of the nasal pyramid, and nasal septum cartilage destruction. Epistaxis, anosmia, and anesthesia of the soft palate, among other symptoms, may occur. (3) the sclerotic stage: this stage is characterized by extensive scarring and laryngeal, and nasal vestibular stenosis in severe cases^10^, ^11^.

Scleroma is an opportunistic disease in immunosuppressed patients and has many differential diagnoses, such as specific granulomatous diseases caused by bacteria (tuberculosis, actinomycosis, syphilis and leprosy), by fungi (histoplasmosis, blastomicosis, paracoccidioidomycosis), by other parasites (mucocutaneous leishmaniasis), systemic diseases (sarcoidosis and Wegener's granulomatosis), and neoplasms (verrucous carcinoma)^12^.

The patient in our case had a tumor that extruded from the right nare. It was typically a hypertrophic stage lesion. The first diagnosis, however, was of a neoplasm, although granulomatous diseases had not been excluded.

Given the emotional situation of the patient and based on the physical exam and radiographic data, we decided for surgery even without a preoperative biopsy. The complete specimen was sent to pathology. Frequently in these cases biopsies do not provide a final diagnosis, due to secondary contamination and insufficient material.

Histopathology defines the diagnosis based on finding Mikulicz cells and degenerated plasmocytes in Russel bodies, described in 1877 by Johann Von Mikulicz^13^. The etiological agent is Klebsiella rhinoscleromatis, a Gram-negative facultative intracellular encapsulated bacillus, a member of the Enterobacteriaceae family identified by Von Frisch in 1882^14^.

Mikulicz cells are clear cytoplasm vacuolated histiocytes containing the bacillus. Factors involved in transforming histiocytes into Mikulicz cells are unknown^9^.

Antibiotic treatment is used as a single treatment to eradicate the infection mostly in the catarrhal stage, or as ancillary treatment in other stages of the disease to reduce mortality and avoid complications. Drug treatment may be combined with surgery in cases with granulomatous lesions or scarring stenosis. Many antibiotics have been used to treat rhinoscleroma. Streptomycin has severe side effects, especially on the vestibular system; also resistance to this drug has developed in a number of countries. Tetracycline requires a prolonged course of treatment and also has significant adverse effects. Rifampicin provides good results in the treatment of rhinoscleroma, but requires effective monitoring of toxicity. Trimethoprim-sulphamethoxazole may be effective and is widely used in Third World countries. Recent studies have shown that the quinolones, ciprofloxacin and fluoroquinolone readily penetrate the tissues and are clinically effective^3^.

## FINAL COMMENTS

Scleroma is a better name for this clinical entity, as it affects both the nasal cavity and the remaining respiratory tract. It is a rare condition in our country and frequently is difficult to diagnose. This is due to the disease stages, where patients may present with non-specific rhinitis symptoms, signs of granulomatous proliferation or scarring.

When the disease progresses with proliferation, it may simulate a tumor, as in our patient. At a later stage, scarring may produce retraction in any part of the respiratory tract.

Material should be adequate for histopathology. A cell infiltrate containing lymphocytes and plasmocytes including Mikulicz cells and Russel bodies is highly suggestive of scleroma. The differential diagnosis should be made with granulomatous diseases, leprosy and tuberculosis, all of which may be identified with specific histopathological techniques.

Isolating Klebsilela Rhinoescleromatis using special culture media is not always possible in these cases.

The correct diagnosis is given based on histopathological findings and the clinical history, which includes information on living conditions and the work environment.
